# WEIGHT REGAIN AND THE METABOLIC PROFILE OF WOMEN IN THE POSTOPERATIVE PERIOD OF BARIATRIC SURGERY: A MULTIVARIATE ANALYSIS

**DOI:** 10.1590/0102-672020230037e1755

**Published:** 2023-08-14

**Authors:** Andréia Lira Santos, Silvia Alves da Silva, Larissa Maria Cavalcante-E-Silva, Luís Henrique de Albuquerque Leão, Lucas Ribeiro Coutinho, Fernando de Santa Cruz Oliveira, Álvaro Antonio Bandeira Ferraz

**Affiliations:** 1 Universidade Federal de Pernambuco, Post Graduate in Surgery – Recife (PE), Brazil; 2 Universidade Federal de Pernambuco, Nutrition Department – Recife (PE), Brazil; 3 Universidade Federal de Pernambuco, Faculty of Medicine – Recife (PE), Brazil; 4 Hospital dos Servidores do Estado, General Surgery Service – Recife (PE), Brazil; 5 Universidade Federal de Pernambuco, University Hospital, General Surgery Service – Recife (PE), Brazil.

**Keywords:** Bariatric surgery, obesity, Body weight changes, Cirurgia bariátrica, Obesidade, Alterações do peso corporal

## Abstract

**BACKGROUND:**

Weight regain in the postoperative period after bariatric surgery is directly related to the relapse of preoperative comorbidities and a negative impact on the patients’ biochemical profile.

**AIMS:**

To assess the metabolic impact of weight regain on preoperative comorbidities and on patients’ biochemical profiles, in order to show the impact of the complications on the metabolic outcomes of bariatric surgery.

**METHODS:**

A retrospective study was carried out with 75 women in the late postoperative period of bariatric surgery who presented pathological weight regain (≥20% of the maximum weight loss). Data of interest consisted of glycemic, lipid, and inflammatory profile measurements at three different moments of evaluation: preoperative period, at the weight nadir (minimum weight), and after weight regain. A multivariate analysis was performed.

**RESULTS:**

The mean age was 46.39±12.09 years. Preoperative body mass index was 40.10±4.11 kg/m^2^. There was an overall increase of 3.36 points in the mean body mass index between the nadir and after regain: from 26.30±3.9 kg/m^2^ to 29.66±4.66 kg/m^2^. The mean time to reach the nadir was 18±7.6 months, with an average percentage of excess weight loss of 91.08±11.8%. The median time for pathological weight regain was 48 months, and the mean regain amongst the sample was 8.85±5.65 kg. There was a significant correlation between pathological weight regain and levels of insulin (r=0.351; p<0.011), C-peptide (r=0.303; p<0.011), C-reactive protein (r=0.402; p<0.001), and vitamin D (r=-0.435; p<0.001), the last two being the most influenced by the percentage of weight regained.

**CONCLUSIONS:**

The pathological weight regain in the postoperative period of bariatric surgery results in losses in the patients’ metabolic and inflammatory profiles. However, the biochemical benefits are sustained up to the preoperative levels of the parameters analyzed.

## RESUMO

**RACIONAL:**

Reganho de peso no pós-operatório de cirurgia bariátrica está diretamente relacionado à recidiva das comorbidades pré-operatórias e a um impacto negativo no perfil bioquímico desses pacientes.

**OBJETIVOS:**

avaliar o impacto metabólico do reganho de peso nas comorbidades pré-operatórias e no perfil bioquímico desses pacientes, a fim de mostrar o impacto das complicações nos desfechos metabólicos finais da cirurgia bariátrica.

**MÉTODOS:**

Estudo retrospectivo que analisou 75 mulheres no pós-operatório tardio de cirurgia bariátrica que apresentaram reganho patológico de peso (=20% do máximo de peso perdido). Foram coletados dados referentes às medidas dos perfis glicêmico, lipídico e inflamatório em três momentos distintos de avaliação: no pré-operatório, no nadir de peso (menor peso) e após o reganho ponderal. Foi realizada uma análise multivariada.

**RESULTADOS:**

A idade média foi 46.39±12.09 anos. IMC médio pré-operatório foi 40.10±4.11 kg/m^2^. Houve um aumento de 3,36 pontos no IMC médio entre o nadir e após reganho: de 26.30±3.9 Kg/m^2^ para 29.66±4.66 Kg/m^2^. O tempo médio para atingir o nadir foi de 18±7.6 meses, com uma %PEP de 91.08±11.8%. O tempo médio para o reganho patológico foi de 48 meses, e a média de reganho foi 8.85±5.65 kg. Houve correlação significativa entre o reganho patológico e os níveis de insulina (r=0.351; p<0.011), peptídeo C (r=0.303; p<0.011), proteína C reativa (r=0.402; p<0.001) e vitamina D (r=-0.435; p<0.001), sendo os dois últimos os mais influenciados pela porcentagem de reganho de peso.

**CONCLUSÕES:**

O reganho de peso patológico no pós-operatório de cirurgia bariátrica resulta em prejuízos ao perfil metabólico e inflamatório dos pacientes. No entanto, os benefícios bioquímicos perduram em relação aos níveis pré-operatórios dos parâmetros analisados

## INTRODUCTION

Obesity is one of the main risk factors associated with morbidity and mortality in adults, being related to 63% of all deaths caused by chronic noncommunicable diseases (CNDs)^3,15^. In this sense, in situations of non-successful clinical treatment of obesity and/or its associated comorbidities, bariatric surgery (BS) figures as the most effective therapeutic tool to achieve and maintain long-term weight loss^3,16,17^.

Therefore, the postoperative weight regain is of significant concern in BS, since rates of up to 67% of pathological weight regain (>20% of the maximum weight loss) five years following the procedure have been reported in the literature^10^. In addition to psycho-emotional impacts, weight regain in the postoperative period after BS directly causes the return, or even worsening, of preoperative comorbidities and a negative impact on the patients’ biochemical profile^5,7,16,22^.

In this scenario, there is a higher incidence of weight regain in women. This is particularly alarming due to the higher prevalence of obesity in this population and, consequently, the predominance in the absolute number of BS performed^3,9,15–17^. Thus, it is essential to assess the metabolic impact of weight regain on preoperative comorbidities and on the biochemical profile of these patients in order to show the impact of this complication on BS metabolic outcomes.

## METHODS

### Study design

This study consists of a retrospective cohort carried out through the collection of secondary data in medical records which included patients undergoing sleeve gastrectomy (SG) or Roux-en-Y gastric bypass (RYGB) at our center between 2010 and 2020. Female patients aged ≥18 years, who presented anthropometric and laboratory data referring to the three different moments of the research (preoperative, nadir of weight, and weight regain) and those who presented weight regain were included. Pregnant patients, patients who used chronic corticosteroids, those on antiretroviral therapy, patients who had undergone another type of surgical intervention of the gastrointestinal tract, and those who missed follow-ups were excluded from the study.

The variables analyzed in the present study were collected at two times: preoperatively and postoperatively. The first was up to 30 days before surgery The latter was divided into the nadir of the postoperative weight loss curve and into standard follow-up periods defined at 3, 6, 9, 12, and 18 months after surgery, with a sequence of annual records after this period. The preoperative clinical, biochemical, anthropometric, and demographic variables analyzed were age, use of vitamin supplementation, weight, height, body mass index (BMI), total cholesterol (TC), high-density lipoprotein (HDL), low-density lipoprotein (LDL), triglycerides (TG), fasting glucose (FG), glycated hemoglobin (HbA1c), insulin, C-peptide, C-reactive protein (CRP), iron, ferritin, zinc, albumin, and vitamin D.

Based on anthropometric data, the following variables were determined: weight regain in kilograms (WR) and percentage of weight regain (%WR). The regain was considered based on the observed weight regain from the nadir and classified as clinically significant when weight increased ≥20% of the maximum weight loss (weight regain – nadir weight)×100 / (preoperative weight – nadir)^9^. Regarding the analysis of the weight curve of patients in the postoperative period, two main points of analysis were defined: weight at the nadir (when a lower weight was achieved after surgery) and regained weight (when weight gain was detected in relation to the nadir).

### Ethical approval

All procedures performed in this study involving human participants were in accordance with the ethical standards of the institutional research committee and with the 1964 Declaration of Helsinki and its later amendments, or comparable ethical standards. This research project was approved by the Ethics Committee of our institution under CAAE no. 98850718.2.0000.5208.

### Statistical Analysis

Statistical analysis was conducted using the Statistical Package for the Social Sciences (SPSS), version 13.0, and Stata, version 14. The sample was characterized by absolute frequencies, percentages, and 95% confidence intervals (CI). To test the normality of quantitative variables, the Kolmogorov-Smirnov test was used. As the variables presented non-normal behavior, so the medians were compared using the Wilcoxon test.

Correlations between weight regain and biochemical variables were tested using Pearson’s and Spearman’s tests. To test the associations of general and metabolic variables with weight regain, Pearson’s chi-square, Yates’ corrected chi-square, chi-square for trend, and Fisher’s exact tests were applied. All variables with p-value (p)<0.20 in the bivariate analysis were included in the multivariate analysis model, which was performed using Poisson regression with robust variance adjustment.

## RESULTS

A total of 1,391 medical records of women who underwent RYGB or SG between 2010 and 2020 were analyzed. Of those, 197 were considered eligible and 75 were included in the final sample. The mean age was 46.39 years, with a standard deviation (±) of 12.09 years. Preoperative weight and BMI were 104.65±15.28 kg and 40.10±4.11 kg/m^2^, respectively. There was an increase of 3.36 points in the BMI mean considering the mean weight at the nadir and after regaining: from 26.30±3.9 kg/m^2^ to 29.66±4.66 kg/m^2^. The mean time to reach the nadir was 18±7.6 months, with an average percentage of excess weight loss (%EWL) of 91.08±11.8%, among which 96% reached a %EWL of 50%. The median time for weight recovery was 48 months and the frequency was only 22.7% of those who presented %EWL>50%. The mean regain amongst the sample was 8.85±5.65 kg.

The majority of the sample consisted of adult women (85.3%) and only 11 (14.7%) were in the elderly age group. Regarding lifestyle, only 5.4% of women practiced physical activity and 2.7% continued nutritional monitoring postoperatively. About 64% of patients had a significant weight regain. The most used surgical technique was SG, performed on 49 patients (65.3%). The maximum follow-up time was 9.3 years (112 months).

Table 1 shows the association between the percentage of weight regain and the metabolic and nutritional variables studied. There was a statistical difference (p≤0.05) in the CRP analysis (p=0.001), so that the group with weight regain ≥20% had higher levels of this inflammatory marker. In addition, there was also a statistical difference in the analysis of ferritin (p=0.028) and vitamin D (p=0.020), with significantly lower levels recorded in the group with weight regain ≥20%. There was no statistical difference in glycemic parameters or lipid profile.

**Table 1:** Association between the percentage of weight regain and metabolic variables in women undergoing bariatric surgery.

Weight Regain							
	<20%			≥20%			
	n	%	95%CI	n	%	95%CI	p-value
Glucose							0.712╬
Normal	22	34.4	22.9–47.3	42	65.6	52.7–77.1	
High	4	44.4	13.7–78.8	5	55.6	21.2–86.3	
HbA1c							0.202**
Normal	21	42	28.2–56.8	29	58	43.2–71.8	
High	6	24	9.4–45.1	19	76	54.9–90.6	
Insulin							1,000╬
Normal	24	35.3	24.1–47.8	44	64.7	52.2–75.9	
High	0	0	0.00–97.5	1	100	100–100	
C-peptide							0.171**
Low	9	47.4	24.4–71.1	10	52.6	28.9–75.6	
Normal	15	29.4	17.5–43.8	36	70.6	56.2–82.5	
CRP							0.001**
Normal	21	52.5	36.1–68.5	19	47.5	31.5–63.9	
High	3	10.7	2.3–28.2	25	89.3	71.8–97.7	
Total Cholesterol							0.744*
Normal	13	34.2	19.6–51.4	25	65.8	48.6–80.4	
High	14	37.8	22.5–55.2	23	62.2	44.8–77.5	
HDL							0.648╬
Normal	26	37.1	25.9–49.5	44	62.9	50.5–74.1	
Low	1	20	0.5–71.6	4	80	28.4–99.5	
LDL							0.635*
Desirable	12	38.7	21.8–57.8	19	61.3	42.2–78.2	
High	14	33.3	19.6–49.5	28	66.7	50.5–80.4	
Triglycerides							0.195╬
Normal	25	40.3	28.1–53.6	37	59.7	46.4–71.9	
High	2	18.2	2.3–51.8	9	81.8	48.2–97.7	
Iron							0.737╬
Normal	23	36.5	24.7–49.6	40	63.5	50.4–75.3	
Low	3	27.3	6.0–61.0	8	72.7	39.0–94.0	
Ferritin							0.028**
Normal	10	62.5	35.4–84.8	6	37.5	15.2–64.6	
Low	17	28.8	17.8–42.1	42	71.2	57.9–82.2	
Zinc							0.300╬
Normal	18	32.1	20.3–46.0	38	67.9	54.0–79.7	
Low	5	50	18.7–81.3	5	50	18.7–81.3	
Albumin							0.537╬
Normal	25	36.2	25.0–48.7	44	63.8	51.3–75.0	
Low	0	0	0.00–84.2	2	100	100–100	
Vitamin D							0.020**
Desirable	12	57.1	34.0–78.2	9	42.9	21.8–66.0	
Low	7	21.9	09.3–40.0	25	78.1	60.0–90.7	

95%CI: 95% confidence interval; CRP: C-reactive protein; HDL-c: high- density lipoprotein; LDL-c: low-density lipoprotein.

* Pearson’s χ² test;

** Pearson’s χ² test with Yates correction; ┼χ² test for trend;

╬ Fisher’s exact test.

Table 2 presents the medians of the metabolic variables studied herein at different phases of the study. With the exception of zinc, ferritin, and albumin, all other serum markers changed significantly (p≤0.001) when comparing the preoperative period to the nadir, showing a positive metabolic response to weight loss. Comparing the same markers at the nadir and after weight regain, there were important changes. There was a statistically significant difference in the values of FG, HbA1c, insulin, C-peptide, TC, and ferritin (p<0.05), illustrating the negative impact of weight regain on such measurements. When correlating these same variables in the preoperative period and in recurrence, all markers, with the exception of zinc, iron, albumin, and vitamin D, kept a statistically significant difference (p<0.001). This suggests a possible maintenance of the metabolic and nutritional benefits achieved in the postoperative period despite weight regain.

**Table 2:** Comparison between the metabolic and nutritional profile in the preoperative period, nadir and regain of women undergoing bariatric surgery.

	Preoperative		Nadir		Regain	
	Median	P25–P75	Median	P25–P75	Median	P25–P75
Glucose	94.00*	86.0–104.0	82.00	78.0–86.2	85.00┼	81.0–90.0
HbA1c	5.70*	5.4–6.1	5.40	5.0–5.6	5.50┼	5.1–5.8
Insulin	20.00*	13.0–30.0	5.00	3.0–8.0	7.00┼	5.0–11.0
C-peptide	3.14*	2.2–4.2	1.60	1.2–2.2	1.80┼	1.4–2.6
CRP	1.47*	0.6–5.8	0.86	0.2–1.7	0.50	0.1–2.5
Total Cholesterol	202.0*	175–225	179	156–216	190.0┼	158–215
HDL	47.00*	39.0–53.6	57	48.0–63.0	55.1	48.0–65.0
VLDL	26.00*	24.4–36.0	17.2	13.0–137	16.20	13.4–25.1
LDL	123.40*	99.2–142.0	108	79.4–137	109.3	79.7–136
Triglycerides	138.00*	113–192	82.00	62.0–121	78.00	55.5–127
Iron	82.00*	57.0–96.0	92.5	69.2–110	88.5	61.0–114
Ferritin	85.00	46.3–138.0	73	30.8–155	30.00┼	10.2–77.0
Zinc	81.10	70.2–89.1	82.95	62.5–90.5	89.15	75.0–92.8
Albumin	4.10	4.0–4.4	4.22	4.0–4.5	4.30	4.1–4.5
Vitamin D	23.70*	19.0–33.2	28.8	14.2–34.2	27.10	21.1–34.4

Wilcoxon test.

* Statistical significance at 5% in the comparison between preoperative and the nadir weight;

┼ Statistical significance at 5% in the comparison between the nadir and regain weight

Table 3 indicates the correlation between weight regain in absolute values and the metabolic and nutritional variables postoperatively. There was a significant correlation (p<0.05) only for the variables such as insulin (r=0.351; p<0.003), C-peptide (r=0.303; p<0.011), CRP (r=0.402; p<0.001), and vitamin D (r= -0.435; p<0.001), the latter in an inverse relationship.

**Table 3:** Correlation between weight regain in kilograms, metabolic and nutritional variables at postoperative time in women undergoing bariatric surgery.

Weight Regain (kg)			
	r	R²	p-value
Glucose	-0.006	0.000	0.959**
HbA1c	0.066	0.004	0.574**
Insulin	0.351	0.123	0.003**
C-peptide	0.303	0.092	0.011*
CRP	0.402	0.162	0.001**
Total Cholesterol	0.115	0.013	0.325*
HDL	-0.106	0.011	0.368*
VLDL	0.193	0.037	0.155*
LDL	0.140	0.020	0.237*
Triglycerides	0.130	0.017	0.274**
Iron	-0.100	0.010	0.396*
Ferritin	0.015	0.000	0.897**
Zinc	0.061	0.004	0.628**
Albumin	-0.088	0.008	0.464**
Vitamin D	-0.435	0.189	0.001*
Postoperative Time (months)	0.458	0.2098	0.000
Weight gain (kg)	0.458	0.2098	0.000

r: correlation coefficient; R²: coefficient of determination; CRP–c: C-reactive protein; TC: Total Cholesterol; HDL: high-density lipoprotein; VLDL: very low-density lipoprotein; LDL: low-density lipoprotein.

* Pearson correlation rest;

** Spearman correlation test.

The correlation coefficient was also applied to assess the relationship between metabolic and nutritional variables, weight regain (in kilograms) and postoperative time (in months) in the sample of women studied (Table 4). The results presented in Table 4, show a relevant and positive correlation between recovery and time (r=0.458; p=0.000) and a negative correlation between time and serum levels of vitamin D (r= -0.488; p=0.000).

**Table 4:** Correlation between postoperative time, metabolic and nutritional variables and weight regain in kilogram of women undergoing bariatric surgery.

Postoperative time (months)			
	r	R²	p–value
Glucose	0.040	0.0016	0.736**
HbA1c	0.182	0.0331	0.117**
Insulin	0.051	0.0026	0.680**
C-peptide	0.109	0.0119	0.370*
CRP	0.153	0.0234	0.213**
Total Cholesterol	-0.035	0.0012	0.766*
HDL	0.010	0.0001	0.934*
VLDL	0.001	0.0000	0.996*
LDL	-0.038	0.0014	0.751*
Triglycerides	-0.037	0.0014	0.754**
Iron	-0.182	0.0331	0.120*
Ferritin	-0.050	0.0025	0.672**
Zinc	0.193	0.0372	0.120**
Albumin	-0.215	0.0462	0.072**
Vitamin D	-0.488	0.2381	0.000*
Weight gain (kg)	0.458	0.2098	0.000

r: correlation coefficient; R²: coefficient of determination; CRP: C-reactive protein; HDL: high-density lipoprotein; VLDL: very low-density lipoprotein; LDL: low-density lipoprotein.

* Pearson correlation test;

** Spearman correlation test.

A multivariate analysis was performed using Poisson regression with robust variance adjustment applied to variables that presented p<0.20 in the bivariate analysis. After the test, the results revealed that the most correlated markers (Table 5) with the percentage of weight regain were vitamin D, reduced about 1.33 times (p=0.000), followed by CRP, which was 1.30 times higher (p=0.000) in weight regaining above 20% of the maximum weight lost.

**Table 5:** Crude and adjusted prevalence ratios of weight regain in percentage according to metabolic variables of women undergoing bariatric surgery.

	Raw analysis		Adjusted analysis			Raw analysis		Adjusted analysis*		
	PR	95%CI	PR	95%CI	p**	PR	95%CI	PR	95%CI	p--**
CRP										
Normal	1.00		1.00		0.005	1.00		1.00		
High	1.34	1.17–1.54	1.35	1.16–1.57	0.000	1.28	1.14-1.45	1.30	1.16–1.47	0.000
Vitamin D										
Desirable	1.00	1.04–1.45	1.00			1.00		1.00		
Low	1.17		1.26	1.07–1.48	0.005	1.25	1.05-1.56	1.33	1.16–1.52	0.000

PR: prevalence ratio; 95%CI: 95% confidence interval; CRP: C-reactive protein.

** Poisson regression with robust variance adjustment.

## DISCUSSION

The evolution of the anthropometric and biochemical variables between the preoperative and the nadir weights shows the positive impact of BS on weight loss and the patients’ metabolic profiles, corroborating the resolute character and the efficiency of this method^3,6,16,17^. However, ensuring positive long-term outcomes is challenged by the incidence of pathological weight regain and by the impact of this complication on the patients’ metabolic and inflammatory profiles^18^.

In the present study, despite the satisfactory %EWL found in the sample, there was no maintenance or progression of body weight loss, so that a median time of 48 months for weight recovery was observed. This finding is in accordance with data previously reported in the literature, which pointed to a stabilization of weight from the second year after surgery, with a significant increase from 48 months onwards^2,11,16^.

In addition, the mean weight regain found in the present work, although in line with the data provided in the literature, was verified in a shorter time interval, which reinforced the magnitude and precocity of the results found^3,8^. This finding can be explained by the postoperative behavior of patients included in this study since, in the postoperative period, only 5.4% of them practiced physical activity and 2.7% were accompanied by a nutritionist, showing the low adherence to the multidisciplinary follow-up^4,8^.

In order to analyze the metabolic and inflammatory impacts even after significant weight regain (average of 56.5%), Lopes et al.^14^ conducted an observational study in which patients with weight regain in the late postoperative period of BS were compared to a group of patients who had never been submitted to BS with a compatible BMI (41.5 vs 40.7 kg/m^2^)^14^. The authors showed that, on average, individuals in the late follow-up of RYGB had significantly lower values of glycemic parameters, such as FG and HbA1c, and higher levels of HDL-c compared to the non-operated group as well as lower rates of dyslipidemia, diabetes mellitus type 2, and abdominal circumference. Similarly, Adams et al.^1^ conducted a 12-year follow-up prospective study which found persistence of metabolic advantage in patients submitted to BS in comparison with those who had never been operated on despite weight regain^1^. Namely, patients who return to the “obese” status after BS still presents better glycemic and lipid profile than those with obesity never submitted to BS.

In the present analysis, the impact of postoperative weight regain did not lead to a return to preoperative levels. Instead, it acted as a warning factor for the return of or maintenance of pre-existing comorbidities. The comparison of biochemical data between the nadir and after weight regain points to significant increases in metabolic markers such as HbA1c, FG, insulin and CT, in addition to a decrease in vitamin D and ferritin levels. Regarding the impact of weight regain, there was no statistical difference for such metabolic variables between patients with pathological (≥20%) and non-pathological (<20%) regain. This fact seems to support the long-term benefits of BS despite weight regain since, compared to preoperative rates, there was a statistically significant reduction in insulin, FG and HbA1c levels, and an increase in HDL-c in the late postoperative period.

In addition to biochemical parameters related to glycolipid metabolism, more specific inflammatory markers are also intrinsically associated with weight regain. CRP is an acute phase marker in metabolic stress condition. It depends on both IL-6 and TNF-a, with high serum concentrations related to hypoadiponectinemia and, consequently, to a low anti-inflammatory response^19–21^. Through a retrospective analysis of 163 patients undergoing SG or RYGB, Lautenbach et al.^12^ demonstrated a significant drop in CRP levels associated with accelerated weight loss during the first two postoperative years. In addition, four years after surgery, the authors described the beginning of weight regain in relation to the previous consultation accompanied by a slight, non-significant (p=0.114) increase in serum CRP levels^12^. Lopes et al.^14^ also found anti-inflammatory benefit of RYGB in the late postoperative period in the context of pathological regain through mean values of IL-6, directly related to CRP, considerably higher in the group of individuals without a history of BS and with a similar BMI (p<0.001)^14^.

Similarly, our results showed a significant reduction in the median of serum CRP levels when comparing the late postoperative (regain) to preoperative contexts (0.50 and 1.47, respectively; p<0.001). In addition, there was a relevant discrepancy in the proportion of individuals with high CRP between the pathological and under 20% regained groups (89.3% and 10.7%, respectively; p=0.001). On the other hand, in our study, there was a decrease in CRP concentrations associated with weight gain between the nadir and the late postoperative period (regain), which is in line with the tendency described in the literature. It should be noted, however, that this reduction was not statistically significant and may be related to pre-analytical variables that alter CRP results, such as physiological variations, fasting, use of anti-inflammatory drugs, and physical exercise^13^.

The limitations of the present study are those inherent to a retrospective study. Losses to postoperative follow-up were mainly responsible for the reduction of the sample, which limits the metabolic outcomes conclusions. In addition, serum levels of nutritional markers may have been masked by the continuous use of vitamin-mineral supplementation generally prescribed to these women in the postoperative period.

## CONCLUSIONS

Weight regain is a frequent complication that leads to adverse anthropometric and metabolic outcomes in the postoperative period of BS. In the present study, long-term pathological regain after surgery leads to losses in the patients’ metabolic profile; however, it still maintains superiority in relation to the preoperative status. Still, the correlation observed between weight regain and the increase in serum levels of C-reactive protein and energy metabolism variables points to the return of pro-inflammatory markers and an accumulating metabolic state, which attenuates the benefits initially promoted by BS.
